# Supplementary figures and images for: RosettaSurf—A surface-centric computational design approach
Source: PLoS Comput Biol. 2022 Mar 16;18(3):e1009178. doi: 10.1371/journal.pcbi.1009178 (PMC9015148; doi:10.1371/journal.pcbi.1009178)

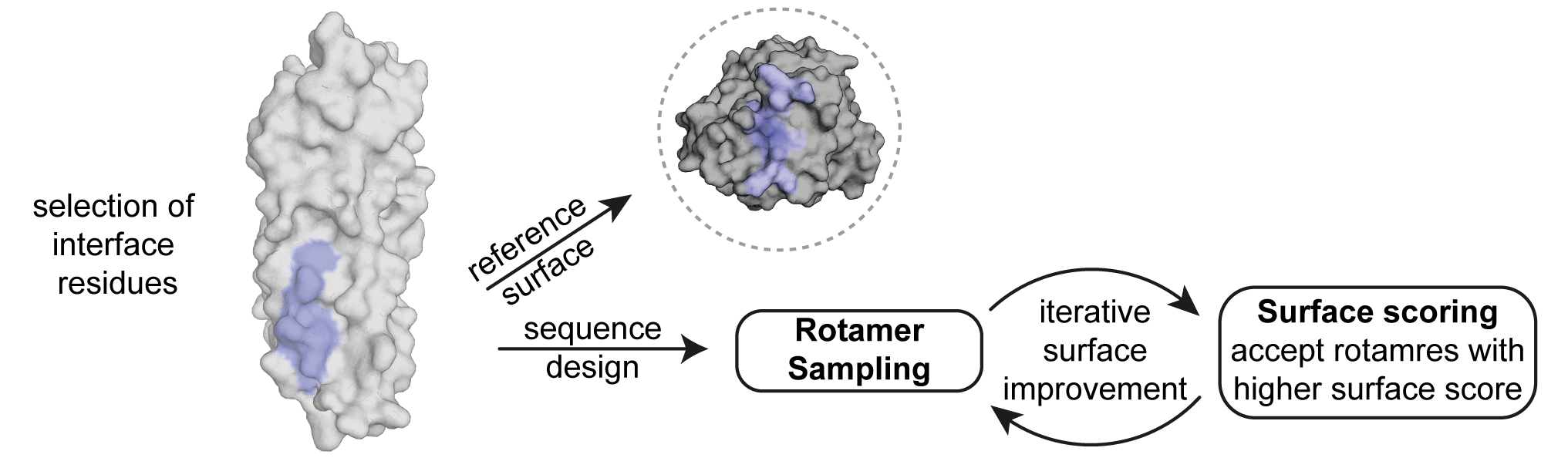

Supplement: S1 Fig — A surface patch is selected on the target protein that will be subjected to mutations for improving surface features. A reference surface is specified and will be used during the design process to guide the introduced mutations. During sequence design, rotamers are sampled in the selected interfaces of the target protein and for each substitution the surface is compared to the reference surface. If mutations improve the surface score, the changes are accepted. During iterative sampling steps of the selected surface patch, the overall surface can be improved. (TIF) [file pcbi.1009178.s001.tif]

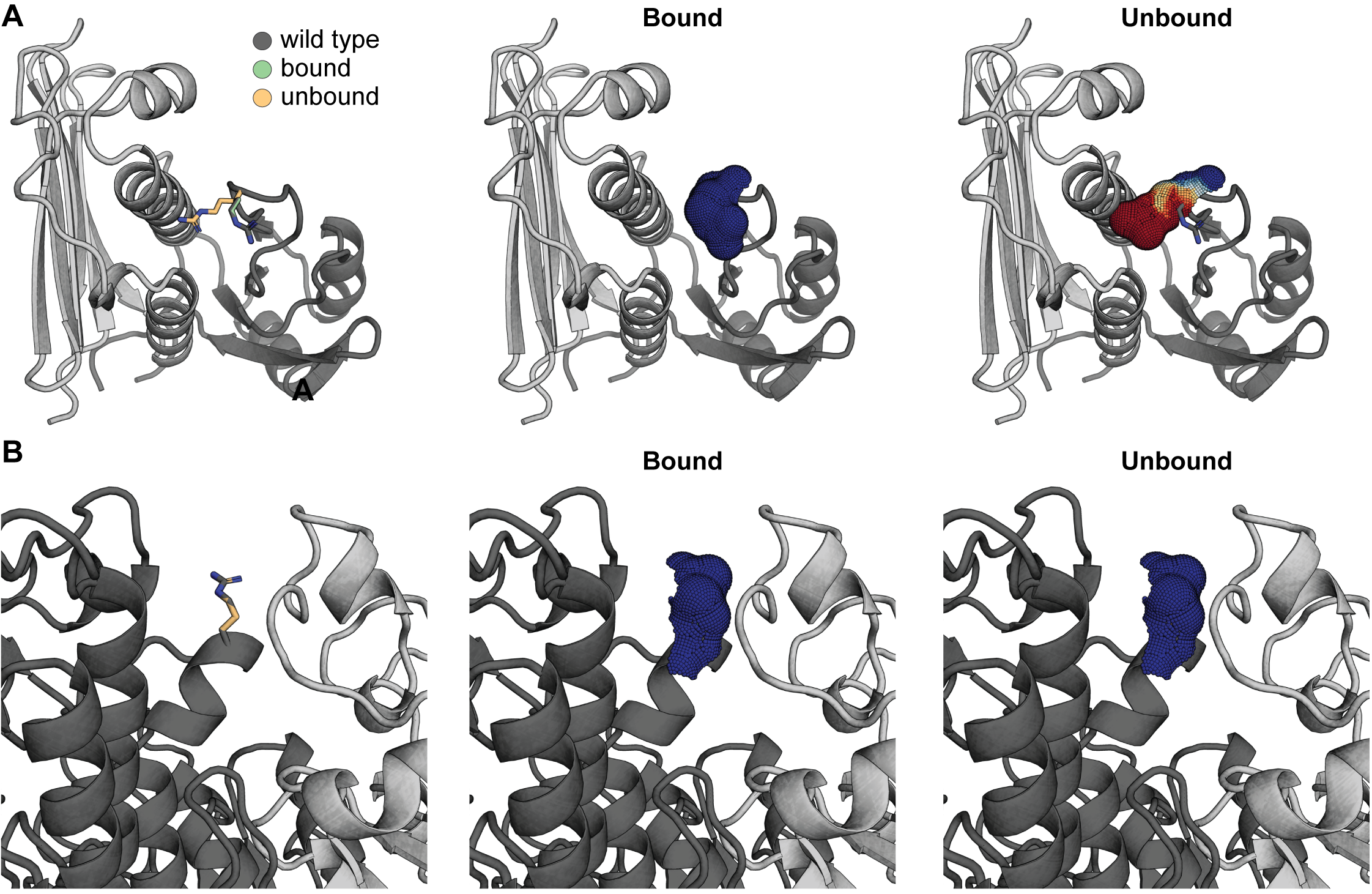

Supplement: S2 Fig — Case study of the recovery of arginine in difficult and easily recoverable benchmark cases. A) Recovery of the exposed arginine residue is unsuccessful in the unbound test case as a non-native rotamer is placed in the structure. The addition of the binding partner limits the accessible rotameric space and allows successful recovery of the amino acid. B) Successful recovery of arginine independent of the presence or absence of the binder as the native rotamer conformation is less exposed. Overall side chain configurations placed in the bound benchmark cases are closer to the native rotamer (mean full-atom RMSD of ~0.4 Å) as compared to the unbound benchmark cases (mean full-atom RMSD of ~2.3 Å). (TIF) [file pcbi.1009178.s002.tif]

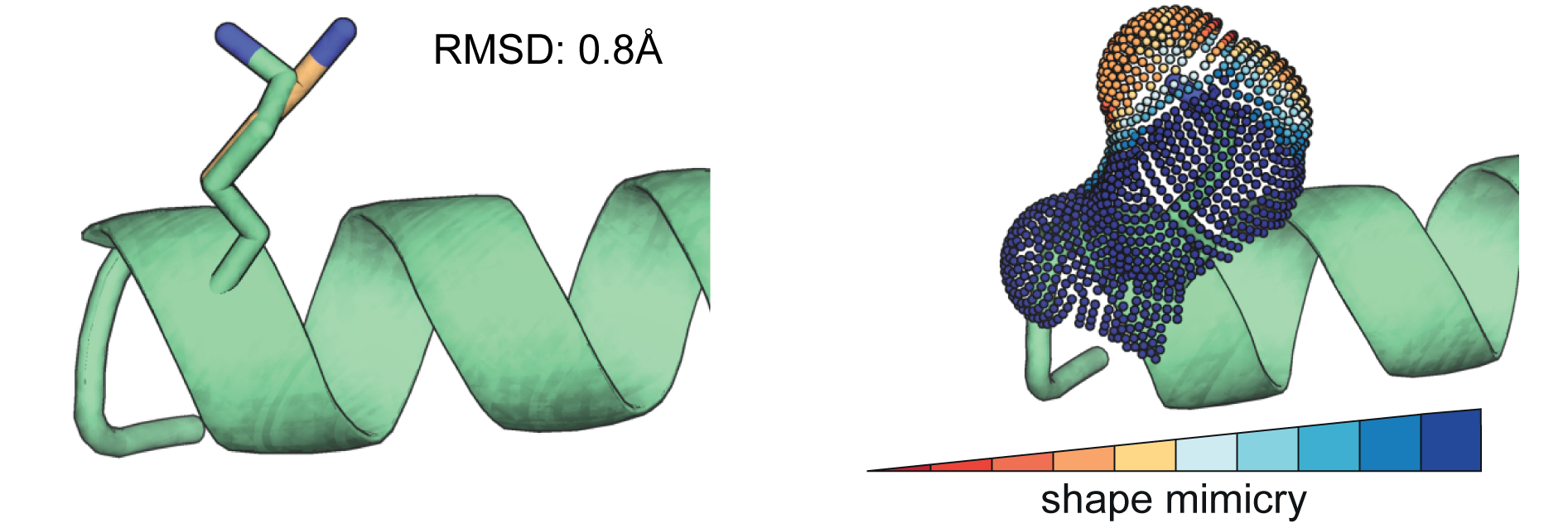

Supplement: S3 Fig — A small rotamer change shown for lysine results in only a 0.8 Å all-atom RMSD change (left). When evaluating both rotamers in terms of surface similarity, the SurfS score can discriminate the local changes. The shape similarity score changes by 0.2 and the electrostatic similarity score by 0.5 units, resulting in an overall SurfS score of 0.989 when comparing both rotamers. The differences are specific for the altered region as shown for shape similarity (right) and outline the modified atom positions. (TIF) [file pcbi.1009178.s003.tif]

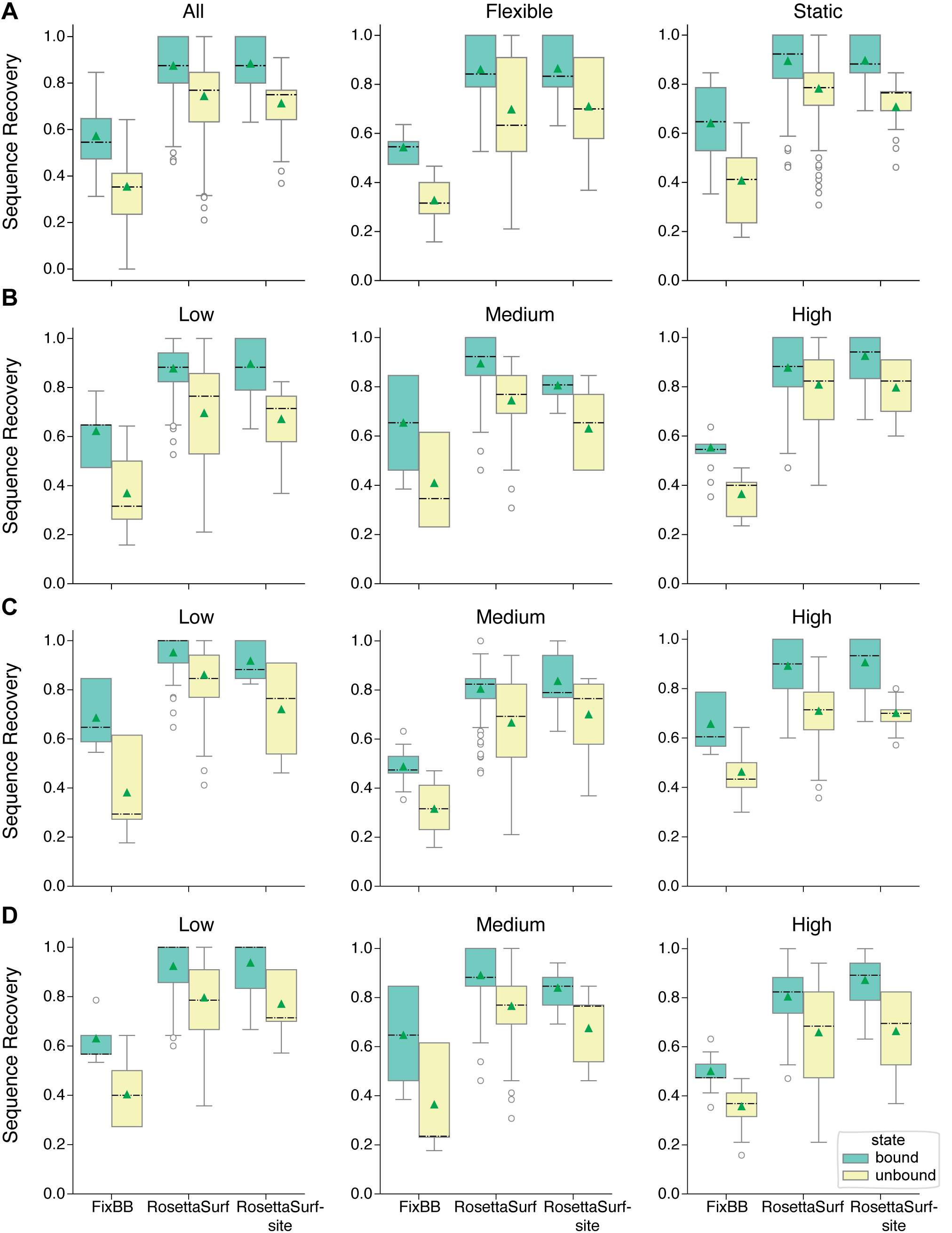

Supplement: S4 Fig — A) Sequence recovery of interfaces from protein complexes grouped by the amount of flexible amino acid side chains. The plot on the left site shows the overall sequence recovery for all complexes combined under the three different design approaches, i.e. FixBB, RosettaSurf, and RosettaSurf-site. The Flexible plot represents the sequence recovery rate of protein complexes whose interfaces consists at least of 40% amino acids with flexible side chains (PDB IDs: 1DFJ, 1EMV, 1PPE, 3MC0). The Static plot contains complexes with less than 40% flexible amino acids in the interface (PDB IDs: 1RV6, 3IDX, 4JLR, 5JDS, 6B9J). Amino acids were considered flexible/static based on Scouras and Daggett’s observations on rotamer dynamics. Accordingly, we assigned R, N, Q, E, H, K, M, and W as flexible and D, I, L, F, P, S, T, Y, V, A, and G as static amino acids. Spearman rank’s correlation analysis shows a weak correlation of sequence recovery rate and rotamer flexibility (ρ = -0.12, p = 5.58e-163), indicating that rotamers with higher conformational flexibility are more difficult to recover. B) Sequence recovery of protein interfaces grouped by the median distance of closest surface points into low (median distance d < 0.5; PDB IDs: 1EMV, 5JDS, 6B9J), medium (median distance 0.5 ≤ d < 0.6; PDB IDs: 1PPE, 3IDX, 4JLR), and high (median distance 0.6 ≤ d; PDB IDs: 1DFJ, 1RV6, 3MC0) groups. C) Sequence recovery of protein interfaces grouped by interface area into low (A < 800 Å2; PDB IDs: 3IDX, 3MC0, 5JDS), medium (800 Å2 ≤ A < 1000 Å2; PDB IDs: 1EMV, 1RV6, 4JLR), and high (1000 Å2 ≤ A; PDB IDs: 1DFJ, 1PPE, 6B9J) groups. D) Sequence recovery of protein interfaces grouped by hydrophobic interface area into low (A < 600 Å2; PDB IDs: 1DFJ, 3MC0, 6B9J), medium (600 Å2 ≤ A < 810 Å2; PDB IDs: 3IDX, 4JLR, 5JDS), and high (810 Å2 ≤ A; PDB IDs: 1EMV, 1PPE, 1RV6) groups. For all benchmark cases, the sequence recovery is reported in the absence (unbound) and presence (bound) of the binder. (TIF) [file pcbi.1009178.s004.tif]

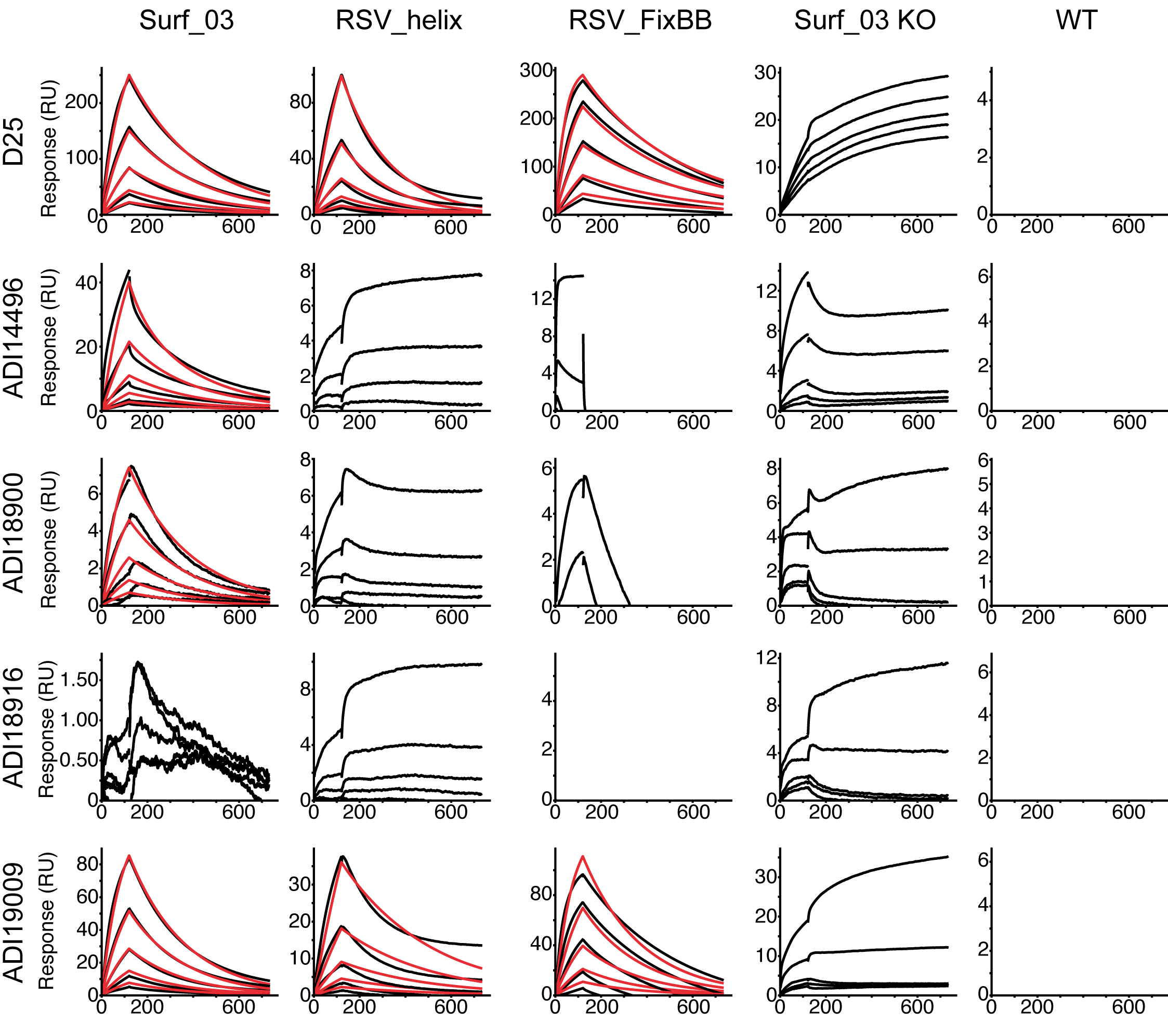

Supplement: S5 Fig — The surface designed protein (Surf_03) demonstrates the broadest binding reactivity, showing measurable interactions with antibodies D25, ADI14496, ADI18900, and ADI19009. In contrast, a design containing solely the helical motif (RSV_helix) of the site 0 epitope interacts only with D25 and ADI19009. Similar results are obtained for a Rosetta-designed variant (RSV_FixBB), binding to D25 and ADI19009. Controls in the form of a KO mutant of design Surf_03 (Surf_03 KO) and the wild type protein (WT) show no measurable binding, indicating that the observed interactions are specific for the designed epitope sites. (TIF) [file pcbi.1009178.s005.tif]
